# *Meriones unguiculatus* serves as a spontaneous primary aldosteronism rodent model

**DOI:** 10.1371/journal.pone.0314943

**Published:** 2025-02-13

**Authors:** Mei You, Zongshi Lu, Bowen Wang, Min Liu, Qing Zhou, Li Li, Dan Tong, Yu Zhao, Hexuan Zhang, Zhongping Bai, Lijuan Wang, Tingbing Cao, Peng Gao, Zhencheng Yan, Zhiming Zhu

**Affiliations:** 1 Department of Hypertension and Endocrinology, Center for Hypertension and Metabolic Diseases, Daping Hospital, Army Medical University, Chongqing Institute of Hypertension, Chongqing, China; 2 Chongqing Institute for Brain and Intelligence, Chongqing, China; District Head Quarter (DHQ) Hospital Charsadda / University of Peshawar, PAKISTAN

## Abstract

**Background:**

Primary aldosteronism (PA) is the most common form of endocrine hypertension. The available animal models of PA rely on gene manipulation, thus fail to duplicate the general pathological process of PA in humans. *Meriones unguiculatus* (MU) has been reported to possess a large size of adrenal gland and an elevated ability to save water. In this study, we aimed to confirm whether MU can serve as an ideal animal model of PA.

**Methods:**

Sprague Dawley rats of the same body weight (SD1) or age (SD2) as MU were used as control groups. Blood pressure and serum aldosterone, renin and electrolyte levels were measured, and the oral salt loading test was used as confirmatory test to compare the inhibition level of the renin angiotensin aldosterone system (RAAS) among the three groups. The expression and distribution of CYP11B2 (aldosterone synthase) were evaluated in the adrenal gland of each group.

**Results:**

MU exhibited typical clinical manifestations of PA, including hypertension, hyperaldosteronism, low renin levels and strong sodium retention and potassium excretion abilities. Compared with control groups, the inhibitory effect of a high-sodium diet on the RAAS was milder in MU, accompanied by significant cardiac dysfunction. The protein expression level and distribution area of CYP11B2 were significantly increased in the adrenal gland of MU.

**Conclusion:**

The current study reveals that MU could serve as an ideal spontaneous PA model. The increased expression and distribution of CYP11B2 stimulate the excessive aldosterone production in a renin-independent manner, leading to a significant increase in blood pressure in MU.

## Introduction

Once considered rare, primary aldosteronism (PA) is now recognized as the most common form of secondary hypertension. The characteristic of PA is the autonomous production of aldosterone from the adrenal gland through a renin-independent mechanism [1]. The inappropriate excess production of aldosterone not only results in sodium and/or volume overload and progressive increases in blood pressure but also leads to more severe cardiovascular, renal, and metabolic outcomes compared with blood pressure-matched essential hypertension [2]. PA accounts for 5–8% of hypertension cases and 11–20% of resistant hypertension cases [3], but the incidence of PA in hypertensive patients may be higher than currently believed, as less than 2% of people at high risk for PA have undergone screening [2]. The measurement of the plasma aldosterone-to-renin ratio (ARR) is currently the most widely used screening method for PA. The confirmatory test is based on the assumption that, under appropriately regulated conditions, complete inhibition of renin production (oral salt loading test and intravenous salt loading test, the two most commonly used tests) or blockade of angiotensin II production (captopril challenge test) should reduce aldosterone production.

Traditionally, sporadic primary aldosteronism is divided into two major subtypes: unilateral and bilateral PA [2]. The pathogenesis of bilateral primary aldosteronism, including bilateral adrenal hyperplasia and idiopathic hyperaldosteronism, is not fully understood [2]. In the past few decades, multiple somatic mutations in driver genes such as ion channels and transport ATPases have been detected in approximately 90% of unilateral aldosterone-producing adenomas (APAs), leading to membrane depolarization, voltage gated calcium channel activation, and increased calcium influx and calcium signaling, resulting in subsequent increased expression of CYP11B2 (aldosterone synthase) and excessive aldosterone production [3]. However, there may be mutations in different driving genes in different nodules of the same APA [4,5] and mutations were also found in the adrenal glands of patients without APA or with secondary nodules expressing CYP11B2, indicating that these mutations may not be initiating factors for PA, but rather compensatory responses to hyperaldosteronism [6,7]. Currently, it remains unclear which factors cause these mutations and whether the previously considered independent pathological steps or subtypes, hyperaldosteronism, adrenal hyperplasia, and APA, are a continuous process of PA. These difficulties are largely attributed to the lack of an animal model that can reflect the development process of spontaneous PA.

Currently, there are two types of family hyperaldosteronism animal models with heterozygous gain-of-function mutations in the calcium channel gene or chlorine channel gene [8,9]. In other PA animal models, excessive aldosterone production is forcibly stimulated by removing background K currents or by activating Gq signaling pathway directly, the downstream of angiotensin Ⅱ [10,11]. These PA animal models have high value for studying the cardiovascular and metabolic consequences of PA or testing the availability of drug interventions, but they cannot naturally replicate the pathological changes in the adrenal gland and therefore cannot represent the exact etiology of PA. Therefore, a more suitable animal model that can naturally develop PA is necessary and urgently needed for a deeper understanding of the pathogenesis of PA.

When we were looking for a reliable animal model of PA, *Meriones unguiculatus* (MU) caught our interest. MU has the abilities to save water and tolerate salt since their natural habitat is semi-desert grasslands [12]. In addition, due to the natural propensity to develop spontaneous proliferative lesions, MU has been applied in the studies of different organs and systems, especially in steroid-responsive organs [13–16]. Moreover, adrenal gland of MU shows a large size compared with other animals [15]. The three zones of the cortex are distinct in adrenal gland of MU, indicating the morphology and, therefore, physiology of adrenal gland of MU are more similar to that of humans [17,18]. Considering these factors, MU may be a potential animal model for PA. However, there have been no reports on whether MU can serve as an animal model for PA so far. Therefore, in this study, we confirmed from physiological and pathological perspectives that MU can indeed be considered as an ideal rodent model for spontaneous PA.

## Methods

### Ethics approval

All experimental procedures were performed in adherence to the National Institutes of Health (NIH) Guide for the Care and Use of Laboratory Animals and in accordance with protocols approved by Laboratory Animal Welfare and Ethics Committee of Third Military Medical University. Sprague Dawley (SD) and MU male rats were purchased from SPF (Beijing, China) Biotechnology Company and housed in cages at a controlled temperature (22 ± 1°C) and relative humidity (55 ± 5%) in a 12-h light/12-h dark cycle at the experimental animal center of Army Medical Center. The rats were sacrificed by overdose of anesthesia (5% isoflurane) and tissue samples were collected for subsequent experiments. We applied SD rats weighing 60–80 g (SD1) as the same weight control, as the body weight of an adult MU is 60–80 g. Similarly, we selected 16-week-old SD (SD2) as the same age control because MU needs to be at least 12 to 16-week-old to be considered sexually mature.

### Oral salt loading test

At 16 weeks of age, the rats were randomly divided into 2 groups and were fed a normal salt diet (0.4% NaCl, Jiangsu Xietong pharmaceutical Bioengineering Co., Ltd, China) or a high-salt diet (8% added NaCl, Jiangsu Madison biomedical Co., Ltd, China) for two weeks. They had free access to food and water.

### Tail-cuff blood pressure measurement

Systolic blood pressure (SBP) and heart rate (HR) were measured by tail-cuff plethysmography (Softron BP-98A system, Japan) before and after two weeks of each diet treatment as previously described [19] for three times and the average value was calculated. The blood pressure of ten rats in each group was measured.

### Serum sample collection

At each time point of the experiment, the rats were anesthetized with 1.5% isoflurane. Blood samples were collected from the fundus orbital vein with a micro blood collection vessel and then transferred into 1.5 mL EP tubes. After 2 hours setting at room temperature, the blood samples were centrifuged (30 minutes, 5000 g), and the supernatants were stored at −80°C until further detection.

### 24-h urine sample collection

The rats in each group were put into the respective metabolic cage (Tecniplast, Italy) for 24 hours. During the experiment, the rats had free access to food and water. The total urine volume was collected. The 24-hour urinary sodium and potassium excretion were measured with an electrolyte detector (XI-921CT Analyzer, Shenzhen China) as previously described [20].

### Serum and 24-h urinary aldosterone measurement

The concentration of aldosterone in rat serum and urine was measured by an UltriMate Thermo 3000 UPLC and TSQ Endura triple quadrupole mass spectrometer (Thermo Fisher Scientific, United States).

### Serum renin measurement

Rat serum renin was measured by an ELISA specific for the detection of Rat Renin 1 (Thermo Fisher Scientific, United States) following the manufacturer’s instructions. The ARR was calculated as the serum aldosterone (ng/dL) concentration divided by the serum renin concentration (pg/mL).

### Steroid measurements

Serum samples were shipped on dry ice to the Shanghai Biotree Biotech Company to perform quantitative detection of high-throughput targets for steroid hormones. Briefly, 16 steroid hormones were detected, and the missing values were filled up by half of the minimum. The final dataset containing the information of sample name, hormone name, and concentration was imported to SIMCA16.0.2 software (Sartorius Stedim Data Analytics AB, Umea, Sweden) for multivariate analysis. Data were scaled and logarithmically transformed to minimize the impact of both noise and high variance of the variables. After the transformations, the heatmap was created using TB tools.

### PET-CT analysis

The uptake and distribution of glucose in vivo were determined by 18F-fluoro-2-deoxy-2-D-glucose (^18^F-FDG) and micro-PET/CT imaging. Briefly, high-resolution PET/CT images of each rat were taken by a small-animal integrated micro-PET/CT scanner (Super Nova ®PET/CT, PINGSENG Health Care) after the injection of ^18^F-FDG through the tail vein. Regions of interest were manually drawn for standard uptake value calculations. All the data were analyzed using the Avatar3 software (PINGSENG Health Care).

### Saline perfusion test

Each rat in the different groups was administered a dose of 1 ml/100 g saline by gavage. Serum samples were collected before and 40 minutes after saline perfusion.

### Echocardiographic evaluation

Echocardiography examination was performed through a high-resolution in vivo micro-imaging system Vevo-770 (Visual Sonics, USA) with a real-time micro visualization scan head of 17.5 MHz. Rats were anesthetized with 1.5% isoflurane and placed in a supine position on a controlled heating pad. Then, their hearts were observed along the short-axis between the papillary muscles and analyzed in M-mode. The systolic and diastolic left ventricular mass (LV mass), percent fractional shortening (% FS) and percent ejection fraction (% EF) were evaluated using M-mode echocardiography.

### Western blot analysis

First, tissue samples were lysed in RIPA lysis buffer with protease inhibitor cocktail tablets (04693132001; Roche, Mannheim, Germany) and phosphatase inhibitor tablets (4906837001; Roche, Mannheim, Germany). The protein concentration was determined using the BCA Protein Assay Kit (23225; Thermo Fisher Scientific). Proteins were separated on 10% SDS–PAGE gels and then transferred to PVDF membranes (IPVH00010; Millipore, USA). The membranes were blocked for 1 h at room temperature in Tris-buffered saline and 0.1% Tween-20 (TBST) containing 5% skim milk and then incubated with primary antibodies in the same buffer at 4 °C overnight as followed: CYP11B2 (ab167413, Abcam, Cambridge, MA, USA), CYP11B1 antibody (sc-377401, Santa Cruz, USA), HSD3B2 antibody (sc-515120, Santa Cruz, USA) and β-actin (66009-1-Ig, Proteintech, USA). After washing and incubation with the appropriate horseradish peroxidase-conjugated secondary antibody (Santa Cruz Biotechnology, USA), the immune complexes were visualized using a chemiluminescence reagent. Western blot results were densitometrically quantified with Quantity One software (Bio-Rad, USA), and the intensity values were normalized to β-actin.

### Histological analysis

Histological analysis was performed using standard techniques. Excised adrenal and heart samples were rinsed in PBS, fixed in 4% paraformaldehyde for 16–24 h, and dehydrated in a series of ethanol washes. Samples were subsequently cleared in xylene and mounted in paraffin. Sections of 3 μm in thickness were cut and stained with hematoxylin and eosin to analyze tissue morphology.

### Immunohistochemical staining

Paraffin sections were labeled with primary antibodies overnight, including rabbit polyclonal CYP11B2 antibody (bs-10161R, 1:100; Bioss), mouse monoclonal CYP11B1 antibody (sc-377401, 1:100, Santa Cruz, USA), mouse monoclonal Dab2 antibody (sc-136964, 1:50; Santa Cruz, USA) and mouse monoclonal β-catenin antibody (sc-7963, 1:50; Santa Cruz, USA). Diaminobenzidine was used as the chromogen, and hematoxylin was used for nuclear counterstaining. Positive-staining areas in the images were quantified using Image-Pro Plus software (version 6.0, Media Cybernetics). Results are presented as the average number of positive-staining area in each HPF.

### Statistical analysis

Quantitative results are expressed as the means ±  SD. The normality and the homogeneity of variance of the data were tested. For data that showed a normal distribution and homogeneity of variance, differences between two groups were compared using a two-tailed Student’s t-test. The differences among three or more groups were analyzed using one-way ANOVA followed by Bonferroni analysis (for data meeting homogeneity of variance) or Tamhane’s T2 analysis (for data demonstrating heteroscedasticity), and a Kruskal-Wallis test plus a post-hoc analysis (Dunn’s multiple comparison test) was used for variables not passing a normality or equal variance test. Two-way ANOVA was applied for data containing two or more independent variables. Graphs were created using Prism 9.0 (GraphPad Software), and statistical analysis was performed with GraphPad Prism. A *P*-value < 0.05 was considered to be statistically significant. No statistical method was used to predetermine the sample size. The data from animal studies were collected using a blinded method. During the final statistical analysis, no data were excluded. A randomization process was performed in grouping rat with the same phenotypes. Animal feedings, treatments and histological analyses were conducted in a single-blinded manner. No blinding was used for the remaining analyses.

## Results

### MU developed spontaneous hypertension and hyperaldosteronism

**Fig 1:**
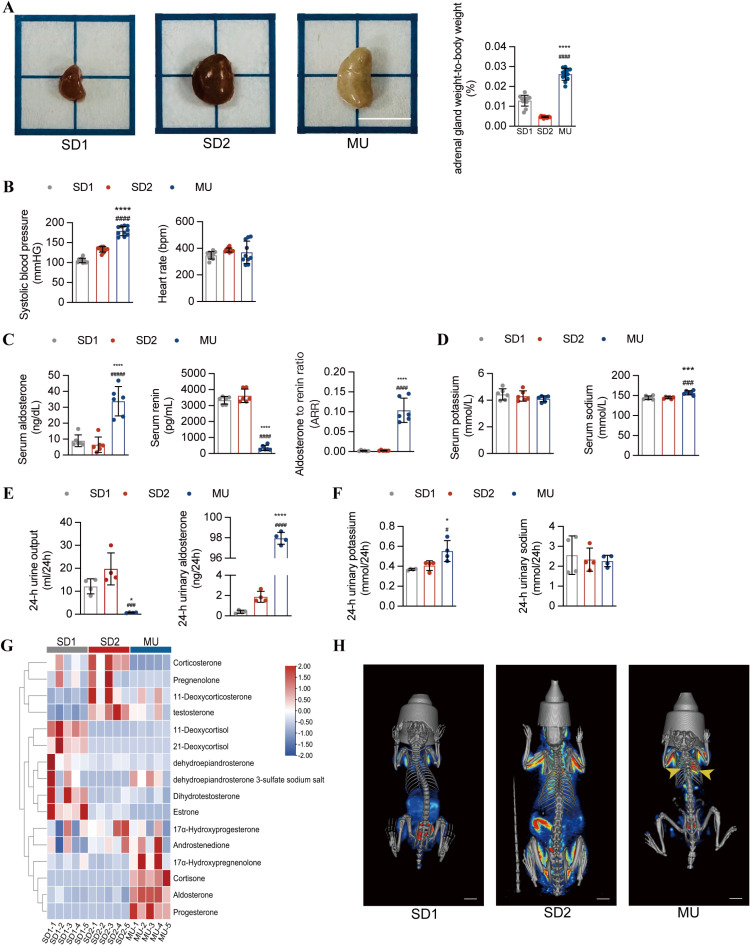
MU rats develop spontaneous hypertension and hyperaldosteronism. **A.** Representative images of the adrenal gland appearance (scale bar =  0.5 cm) and the adrenal gland weight-to-body weight ratio of SD1 (the same body weight control), SD2 (the same age control) and MU rats. **B.** The systolic blood pressure (SBP) and heart rate (HR) of the SD and MU rats were measured by tail-cuff BP plethysmography (n = 10). **C.** Concentrations of aldosterone and renin and the aldosterone-to-renin ratio (ARR) in the serum (n = 6). **D.** Serum concentrations of electrolytes (n = 6). **E.** The amount of 24-h urine output and urinary aldosterone excretion (n = 4). **F.** Twenty-four-hour urinary potassium and sodium excretion (n = 4). **G.** Heatmap of 16 kinds of steroid hormones (n = 5). **H.** Representative PET/CT images of SD and MU rats. The arrow points to the adrenal gland. The results are expressed as the mean ±  SD. *P < 0.05, **P < 0.01, ***P < 0.001, ****P < 0.0001 compared with SD1 group. ^#^*P* < 0.05, ^##^*P* < 0.01, ^###^*P* < 0.001, ^####^*P* < 0.0001 compared with SD2 group.

Compared with the SD1 or SD2, the MU had a higher adrenal gland weight-to-body weight ratio (Fig 1A). The SBP of the MU were significantly higher than those of the SD1 or SD2, but there was no statistically significant difference in heart rate among the three groups (Fig 1B). The serum aldosterone level of MU significantly increased, but the serum renin level was inhibited, leading to an increase in ARR, indicating that the production of aldosterone was not dependent on renin (Fig 1C). Although serum potassium levels were similar among the three groups, serum sodium levels were significantly higher in MU group (Fig 1D). The 24-h urinary aldosterone excretion was also evaluated to reduce the impact of diurnal fluctuations and susceptibility to multiple factors on aldosterone secretion. In addition to a lower 24-h urine output, the 24-h urinary aldosterone excretion of MU group significantly increased by nearly 50–100 times compared with SD1 and SD2, respectively (Fig 1E). Although the 24-h urine sodium concentration of the three groups was similar, the 24-h urine potassium concentration of MU was significantly higher (Fig 1F), indicating that MU indeed has a strong ability to retain sodium, water, and excrete potassium. To further determine the status of steroid hormones in MU, we conducted a metabolomics study targeting steroid hormones and found that compared with SD1 and SD2, the level of aldosterone in MU was significantly increased, accompanied by higher levels of 17α-hydroxypregnenolone, cortisone and progesterone (Fig 1G). Furthermore, adrenal glucose uptake measured by micro-PET/CT was markedly elevated in MU (Fig 1H).

### The inhibitory effect of high-sodium diet on the RAAS was impaired in MU

**Fig 2:**
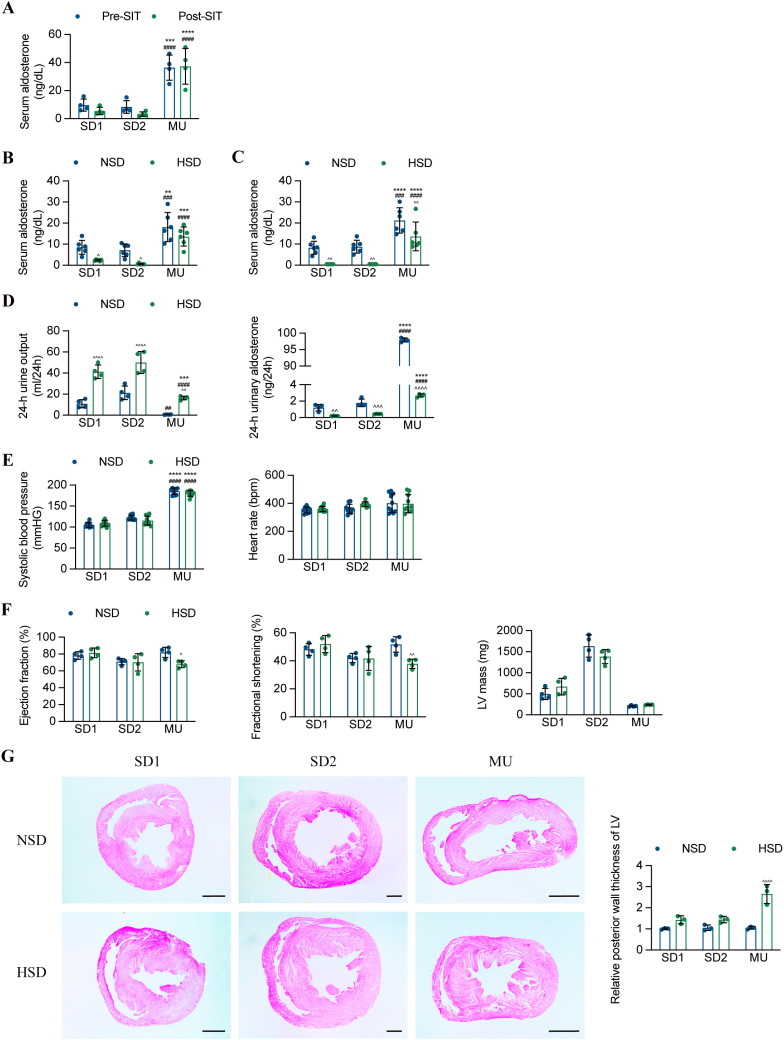
The inhibitory effect of high salt intake on the RAAS is impaired in MU rats. **A.** Serum concentrations of aldosterone before and after saline administration (n = 4). **B-C.** Serum concentration of aldosterone in SD1, SD2 and MU rats fed a normal salt diet (NSD) or a high salt diet (HSD) for 3 days **(**B**)** or 2 weeks **(**C**)** (n = 6). **D.** The 24-h urine output and aldosterone excretion of SD1, SD2 and MU rats fed a NSD or HSD for 2 weeks (n = 4). **E.** The systolic blood pressure (SBP) and heart rate (HR) of SD1, SD2 and MU rats fed a NSD or HSD for 2 weeks were measured by tail-cuff BP plethysmography (n = 10). **F.** The indicated cardiac parameters of SD1, SD2 and MU rats fed a NSD or HSD for 4 weeks (n = 4). **G.** Histological analyses of heart sections from SD1, SD2 and MU rats fed a NSD or HSD for 4 weeks. Heart cross-sections were stained with H&E to analyze hypertrophic growth (scale bar = 200 μm). The results are expressed as the mean ±  SD. *P < 0.05, **P < 0.01, ***P < 0.001, ****P < 0.0001 compared with SD1 group. ^#^*P* < 0.05, ^##^*P* < 0.01, ^###^*P* < 0.001, ^####^*P* < 0.0001 compared with SD2 group. ^*P* < 0.05, ^^*P* < 0.01, ^^^*P* < 0.001, ^^^*P* < 0.0001 compared with NSD group.

The saline infusion test is one of the confirmatory tests of PA [21], but it is difficult to implement in animals. Therefore, in this study, the rats were given saline by gavage instead of infusion. After administration, the serum aldosterone levels of SD1 and SD2 slightly decreased, but there was no corresponding change in MU (Fig 2A). The oral salt loading test is the simplest and most widely used PA confirmatory test in animals [8,11,22,9]. To determine the declining levels of aldosterone induced by high-salt diet, SD1, SD2 and MU were split into 2 groups respectively: (1) normal-salt diet (NSD)(2) high-salt diet (HSD, a well-established model of excessive dietary salt). Following three days of HSD treatment, we found that serum aldosterone significantly decreased only in SD1 and SD2 and even dropped to the lowest detection value in SD2. However, there was no significant change in MU (Fig 2B). Two weeks after HSD treatment, although the serum aldosterone levels of MU significantly decreased within the narrow range, the other two groups showed a decrease of approximately 90% in this parameter, reaching the lowest detection value (Fig 2C). The high salt diet not only significantly increased urine output but also reduced the amount of 24-h aldosterone excretion among the three groups. However, the daily urinary aldosterone excretion of MU was still several times that of SD1 and SD2 (Fig 2D). The blood pressure of MU was still significantly higher than that of the SD1 or SD2 in HSD groups, but there was no statistically significant difference between the NSD and HSD groups (Fig 2E). Experimental evidence suggests that the combination of sodium overload and sustained aldosterone excess has harmful effects on target organs, but exposure to only one of the two factors does not cause cardiovascular toxicity [23,24]. Therefore, the three groups were relatively exposed to HSD for a longer period of 4 weeks. According to the echocardiography evaluation, the cardiac function of MU was significantly impaired, manifested by decreased ejection fraction and shortened fraction (Fig 2F). The histological examination of cardiac slices also showed a significant increase in left ventricular wall thickness in MU after HSD intervention (Fig 2G).

### The expression of CYP11B2 (aldosterone synthase) in the adrenal gland of MU increased, and its distribution range expanded

**Fig 3:**
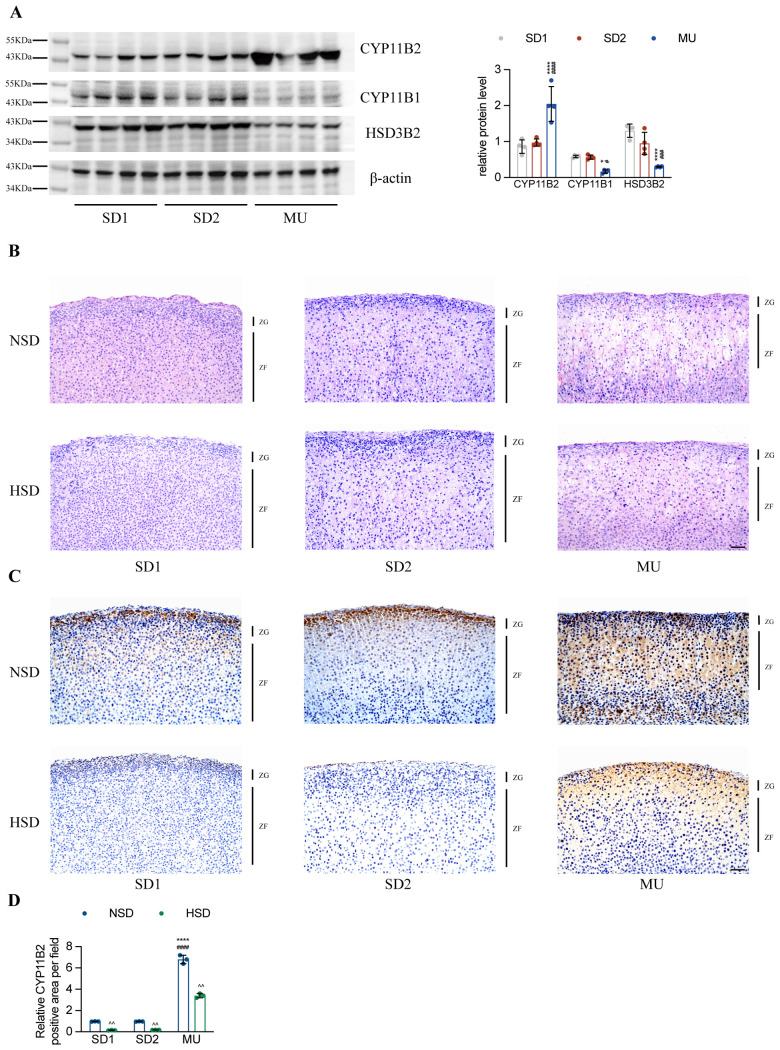
The increased expression and distribution of CYP11B2 in the adrenal glands of MU rats. **A.** Representative western blots showing the levels of CYP11B2, CYP11B1 and HSD3B2 in the adrenal gland. The quantitative results are shown on the right (n = 4). **B.** Histological analyses of adrenal gland sections from SD and MU rats fed a normal or high-salt diet for 2 weeks. Adrenal gland cross-sections were stained with H&E (scale bar = 50 μm). **C.** Representative images showing immunohistochemical staining of CYP11B2 in the adrenal glands. Nuclei were stained with hematoxylin (scale bar = 50 μm). **D.** The relative proportion of CYP11B2-positive areas in the adrenal gland (n = 3). *P < 0.05, **P < 0.01, ***P < 0.001, ****P < 0.0001 compared with SD1 group. ^#^*P* < 0.05, ^##^*P* < 0.01, ^###^*P* < 0.001, ^####^*P* < 0.0001 compared with SD2 group. ^*P* < 0.05, ^^*P* < 0.01, ^^^*P* < 0.001, ^^^*P* < 0.0001 compared with NSD group.

As an important endocrine organ, the adrenal cortex is composed of three distinct zones with different functions [25]. Aldosterone is only produced in the outer layer of the cortex-zona glomerulosa (ZG) because the expression of CYP11B2 is restricted to the ZG. While 11β-hydroxylase (CYP11B1), which catalyzes the last reaction of cortisol production, is expressed in the middle layer of the cortex-zona fasciculata (ZF). In the whole adrenal gland, the expression protein levels of CYP11B2 in MU were significantly increased by nearly 2 to 3 times compared with SD1 and SD2 (Fig 3A), which was correlated with the high circulating aldosterone level in MU. The expression levels of CYP11B1 and 3β-hydroxysteroid dehydrogenase type 2 (HSD3B2), the upstream enzyme of the aldosterone synthetic pathway, were significantly decreased in MU (Fig 3A), which might be due to a negative feedback effect of elevated CYP11B2 expression. According to HE staining, the most obvious difference between the adrenal gland of MU and its controls is the ZF. The ZF comprised larger and foamy cells in MU, and this morphological structure became abated in the HSD group (Fig 3B). Interestingly, zonation of CYP11B2 in MU was out of control, as CYP11B2-positive cells presented not only in the ZG but also in the ZF. The distribution of CYP11B2 decreased after HSD treatment but still beyond the ZG in MU (Fig 3C). These results indicate that CYP11B2-positive ZF cells did not undergo a complete phenotype transition from ZG cells, which may be the direct cause of increased aldosterone secretion.

### The Wnt/β-catenin signaling pathway was activated in the adrenal gland of MU

**Fig 4:**
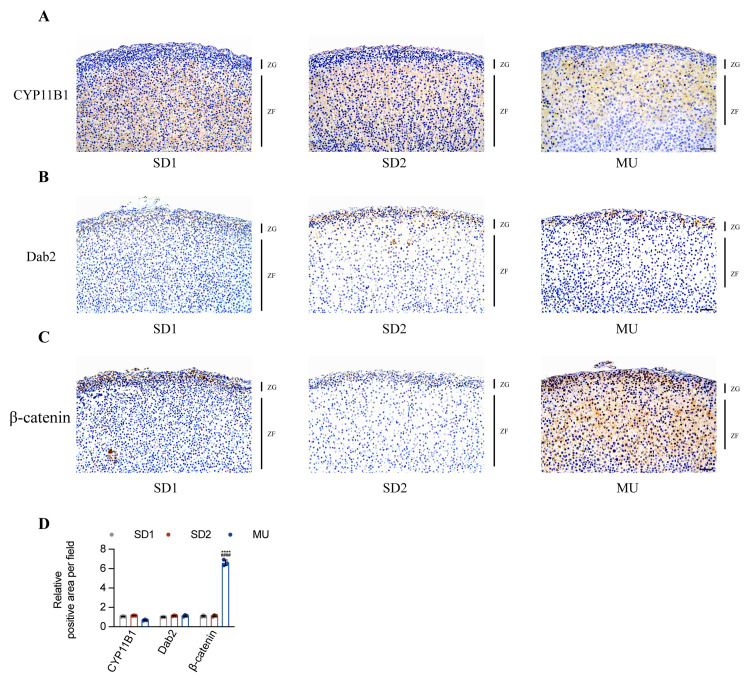
The Wnt/β-catenin signaling pathway is activated in the adrenal glands of MU rats. **A-C**. Representative images showing immunohistochemical staining of CYP11B1 **(A)**, Dab2 **(B)**, and β-catenin **(C)** in the adrenal glands. Nuclei were stained with hematoxylin (scale bar = 50 μm). The quantitative results are shown in **D** (n = 3). *P < 0.05, **P < 0.01, ***P < 0.001, ****P < 0.0001 compared with SD1 group. ^#^*P* < 0.05, ^##^*P* < 0.01, ^###^*P* < 0.001, ^####^*P* < 0.0001 compared with SD2 group.

The expression of CYP11B1, which regulates cortisol synthesis, was restricted to the ZF in all groups (Fig 4A). Highly differentiated ZG cells express Disable-2 (Dab2), a specific ZG marker, but this expression disappears when ZG cells transdifferentiate into ZF cells [26]. Consistent with SD1 and SD2, the expression of Dab2 in MU was only expressed in the ZG (Fig 4B), indicating that there was a clear boundary between ZG and ZF in the adrenal cortex of MU. The Wnt/β-catenin signaling pathway plays an important role in adrenal development, growth, and cell replenishment and is only activated in the ZG under physiological conditions [27,28]. In contrast to SD1 and SD2, the distribution of β-catenin in MU was no longer limited to the ZG but was widely distributed throughout the entire cortex (Fig 4C). The extensive colocalization of β-catenin and CYP11B2 from ZG to ZF suggests that the excessive production of aldosterone may be related to the activation of the Wnt/β-catenin signaling pathway.

## Discussion

This study reveals that MU could serve as an ideal animal model of PA independent of gene manipulation. The elevated expression and dysregulated distribution of CYP11B2 lead to renin-independent hyperaldosteronism, resulting in the spontaneous hypertension in MU. The MU has become increasingly widely used as an experimental animal in biological and biomedical studies alongside other rodents, such as SD rats [15,29–35]. SD rats were applied as controls for MU to study lipid metabolism [32,34], electrolyte homeostasis [36], functional peculiarities of cortical fields [37], as well as animal models of sugar cataracts [38], hyperlipidemia [31], and cerebral ischemia [39]. Therefore, we chose SD rats as the control group in our study. Considering that the production of aldosterone is related to body mass index and age, we selected SD rats of the same body weight and age as the control group.

The manifestations of hypertension, hyperaldosteronism, suppressed renin, elevated urinary aldosterone excretion, sodium overload and potassium overexcretion were highly consistent with the clinical manifestations of PA in humans. High salt diet inactivate adrenal aldosterone synthesis through suppression of renin under normal physiological conditions [11]. However, PA is characterized by pronounced renin-independent aldosterone production. In PA cases, aldosterone will be less inhibited by salt compared with normal individuals. In this study, the slight suppression of RAAS induced by high sodium diet in MU is consistent with the characteristics of PA. CYP11B2 is the enzyme responsible for the final step of aldosterone biosynthesis. PA is caused by dysregulated localization of CYP11B2. Moreover, CYP11B2 positive immunostaining in tumor cells distinguishes an APA from a nonfunctioning adenoma [40]. Therefore, the increased expression and distribution of CYP11B2 in the adrenal gland of MU match the pathogenesis of PA. Therefore, MU serves as an ideal spontaneous PA model. However, it is worth mentioning that due to the lack of posterior connection between the basal artery and carotid artery system of MU, telemetry dynamic blood pressure measurements cannot be performed in this study, which is why MU has become a widely used animal model for cerebral ischemia [39]. Although MU performs well in simulating spontaneous PA, due to the lack of widespread application of gene manipulation and gene delivery systems based on adeno-associated virus in MU, it is still too early to explore the pathogenesis of PA using MU as an animal model [41,42].

The establishment of the centripetal migration model offers another hypothesis of the mechanism of PA. In this model, adrenocortical progenitors give rise to ZG cells, which undergo differentiation and lineage conversion to ZF cells while undergoing centripetal displacement [43]. Moreover, dysregulation of adrenocortical turnover pathways is associated with the development of adrenal tumors [43]. Not only typical APA but also some nodules were reported to be comprised of either small, compact cells (ZG phenotype), or large, foamy, lipid-rich cells (ZF phenotype), or a mixture of the two [1,44], suggesting that both ZG-like and ZF-like cells are capable of expressing the aldosterone synthase enzyme and secreting aldosterone. Unlike other PA models, such as task^-/-^ mice and AS^ + /cre^ hM3Dq mice, where CYP11B2 is widely expressed without hyperplasia or adrenal nodules [11,45], in MU, not only did the protein expression level of CYP11B2 increase but also the distribution of CYP11B2 extended from the ZG to ZF, further proving the correctness of the centripetal migration model. Nevertheless, even though it was reported that the natural occurrence of adrenal cortical carcinoma (ACC) in MU, we did not observe ACC happened in MU possibly due to the relatively short observation time.

Aldosterone-producing cell clusters (APCCs) have been described as large clusters of CYP11B2 cells that do not express Dab2, indicating that APCCs contain cells exhibiting with an intermediate phenotype between ZG and ZF cells [46]. Similarly, we found that the ZF cells expressed both CYP11B2 and CYP11B1 but not Dab2 in the adrenal gland of MU, which means that ZF cells have lost ZG phenotype but retained the ability to secrete aldosterone. Wnt/β-catenin signaling is crucial for the differentiation and maintenance of the aldosterone-producing phenotype in ZG [47]. And activation of Wnt/β-catenin signaling is related to the occurrence of ACC [48]. Our research found that the extensive distribution of β-catenin from ZG to ZF in MU was consistent with CYP11B2. The activation of Wnt/β-catenin signaling can to some extent explain the natural occurrence of ACC in MU, but the exact relationship between the centripetal migration model, activation of Wnt/β-catenin signaling and ACC formation is still unclear.

In conclusion, this study indicates that MU serves as an ideal animal model for PA. In this model, the imbalance of ZG differentiation and lineage transition is the cause of excessive aldosterone production, but it is currently unclear which factor caused this imbalance in the evolution of MU.

## Supporting information

S1_raw images The raw images of western blot. (PDF)

S2 data Relevant data underlying the findings described in manuscript. (XLSX)

## Abbreviations

PA: primary aldosteronism
MU: Meriones unguiculatus
RAAS: renin angiotensin aldosterone system
ARR: aldosterone-to-renin ratio
APA: aldosterone-producing adenoma
ACC: adrenal cortical carcinoma
SD: Sprague Dawley
SBP: systolic blood pressure
HR: heart rate
FS: fractional shortening
EF: ejection fraction
NSD: normal-salt diet
HSD: high-salt diet
ZG: zona glomerulosa
ZF: zona fasciculata
CYP11B1: 11β-hydroxylase
HSD3B2: 3β-hydroxysteroid dehydrogenase type Ⅱ
Dab2: Disabled 2
ZR: zona reticularis
FH: family hyperaldosteronism
ACTH: adrenocorticotropic hormone
APCCs: aldosterone-producing cell clusters.
