# Supplementary figures and images for: Meriones unguiculatus serves as a spontaneous primary aldosteronism rodent model
Source: PLoS One. 2025 Feb 13;20(2):e0314943. doi: 10.1371/journal.pone.0314943 (PMC11824956; doi:10.1371/journal.pone.0314943)

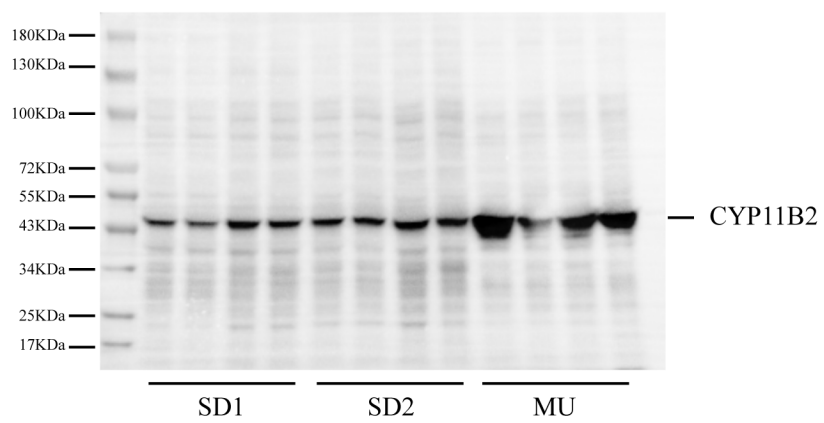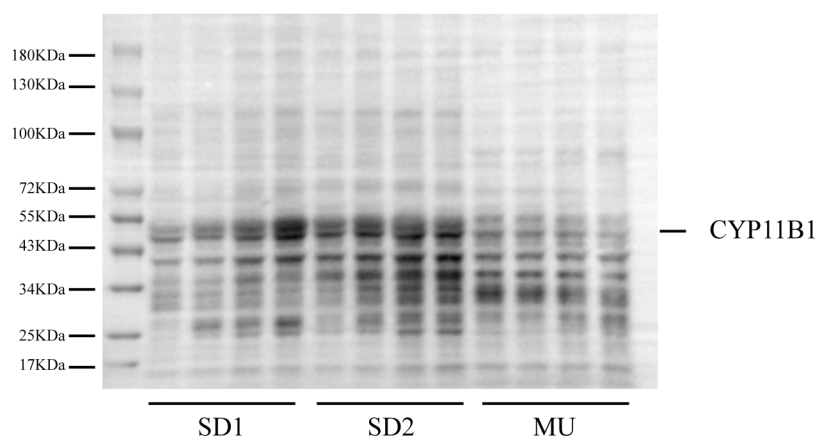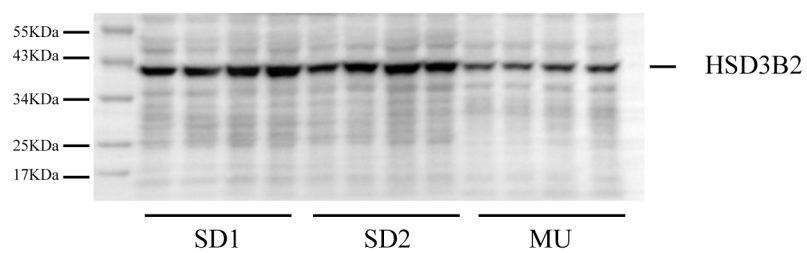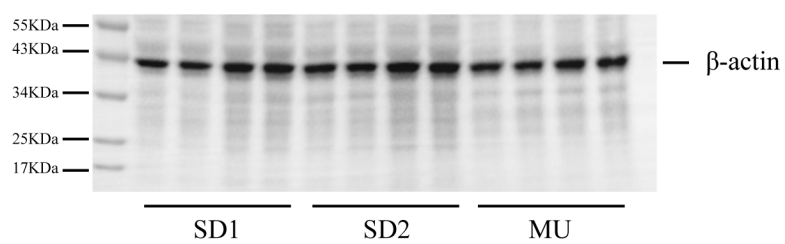

Supplement: S1_raw images — (PDF) [file pone.0314943.s001.pdf]
